# New Insights about the Wnt/β-Catenin Signaling Pathway in Primary Bone Tumors and Their Microenvironment: A Promising Target to Develop Therapeutic Strategies?

**DOI:** 10.3390/ijms20153751

**Published:** 2019-07-31

**Authors:** Geoffroy Danieau, Sarah Morice, Françoise Rédini, Franck Verrecchia, Bénédicte Brounais-Le Royer

**Affiliations:** Université de Nantes, INSERM, UMR1238, Phy-OS, Sarcomes Osseux et Remodelage des Tissus Calcifiés, 44035 Nantes, France

**Keywords:** Wnt/β-catenin, bone sarcoma, bone tumor microenvironment

## Abstract

Osteosarcoma and Ewing sarcoma are the most common malignant primary bone tumors mainly occurring in children, adolescents and young adults. Current standard therapy includes multidrug chemotherapy and/or radiation specifically for Ewing sarcoma, associated with tumor resection. However, patient survival has not evolved for the past decade and remains closely related to the response of tumor cells to chemotherapy, reaching around 75% at 5 years for patients with localized forms of osteosarcoma or Ewing sarcoma but less than 30% in metastatic diseases and patients resistant to initial chemotherapy. Despite Ewing sarcoma being characterized by specific *EWSR1-ETS* gene fusions resulting in oncogenic transcription factors, currently, no targeted therapy could be implemented. It seems even more difficult to develop a targeted therapeutic strategy in osteosarcoma which is characterized by high complexity and heterogeneity in genomic alterations. Nevertheless, the common point between these different bone tumors is their ability to deregulate bone homeostasis and remodeling and divert them to their benefit. Therefore, targeting different actors of the bone tumor microenvironment has been hypothesized to develop new therapeutic strategies. In this context, it is well known that the Wnt/β-catenin signaling pathway plays a key role in cancer development, including osteosarcoma and Ewing sarcoma as well as in bone remodeling. Moreover, recent studies highlight the implication of the Wnt/β-catenin pathway in angiogenesis and immuno-surveillance, two key mechanisms involved in metastatic dissemination. This review focuses on the role played by this signaling pathway in the development of primary bone tumors and the modulation of their specific microenvironment.

## 1. Primary Bone Tumors: Osteosarcoma and Ewing Sarcoma

Osteosarcoma and Ewing sarcoma are the most common primary bone tumors mainly occurring in children, adolescents and young adults. Current standard therapy includes multidrug chemotherapy and/or radiation for Ewing sarcoma, associated with tumor resection. However, the 5-year survival rates have not been improved during the last decades reaching 70–80% for patients with localized forms, but less than 30% in metastatic diseases and patients resistant to initial chemotherapy [1,2,3,4,5].

Osteosarcoma that mainly occurs at the ends of long bones is not associated with any clinical signs except severe pain or spontaneous fracture. Osteosarcoma does not express specific oncogenic markers but exhibits a large number and variety of genetic alterations. Ewing sarcoma is a tumor composed of small undifferentiated round cells that can appear mainly in bones but also in soft tissues in 15% of cases. In contrast to osteosarcoma, Ewing sarcoma is characterized by a chromosomal translocation between the *EWSR1* and *FLI1* genes in 90% of cases, or by the fusion of *EWSR1* with other transcription factors of the E26 Transformation-Specific (ETS) gene family in 10% of cases [6,7,8].

Despite progress in understanding the biology of osteosarcoma and Ewing sarcoma, no targeted therapy could be currently implemented to improve patient survival. Nevertheless, the common point between these two pediatric bone tumors is their ability to deregulate bone homeostasis and remodeling to divert them to their benefit. Therefore, new therapeutic research is moving towards targeting different actors of the bone microenvironment.

## 2. Tumor Microenvironment: Crucial Role in Bone Sarcoma Tumor Growth and Metastatic Progression

It is well established that the bone tumor microenvironment (TME) plays a major role during tumor initiation, progression, and metastatic processes. This very complex and dynamic environment includes different cell types such as osteoblasts, osteoclasts, stromal cells, mesenchymal stem cells, immune or endothelial cells and non-cellular components such as the extracellular matrix compounds [9]. These cells are able to communicate with each other in order to influence their behavior [10]. This microenvironment is able to support bone sarcoma primary development, resistance to chemotherapy [11] and metastatic spread of tumor cells [12,13,14].

The crucial role played by the TME on sarcoma development has been recently highlighted in several studies. As an example, a modification of the transcriptome has been observed in cell lines established from synovial sarcomas compared to initial tumors or directly engrafted tumors in mice, demonstrating the impact of TME on tumor behavior, particularly through interactions with the immune system and stromal cells [15]. The bone TME is also able to affect the behavior and the fate of tumor cells and particularly, their ability to migrate and invade new tissues. Indeed, the development of sarcoma in orthotopic models of xenograft was associated with the establishment of metastases whereas the subcutaneous model did not allow to observe any metastatic spread [16]. These enhanced invasive and migratory capacities have been confirmed in a rat model of prostate carcinoma and related to the acidification of the bone tumor microenvironment [17]. Similarly, Chattopadhyay et al. have observed that solid sarcoma developed differentially when implanted into two different anatomical sites, related to a greater neovascularization and the presence of tumor-associated fibroblasts and tumor-associated adipocytes, which could support sarcoma tumor growth [18].

### 2.1. Hijacking of the Bone Tumor Microenvironment by Bone Sarcoma Cells

One of the main hallmarks of osteosarcoma and Ewing sarcoma is their ability to disrupt bone remodeling in favor of an exacerbated osteoclast-mediated bone resorption, leading to the release of pro-tumoral factors initially embedded in the bone matrix [19,20,21]. It thus establishes a vicious cycle between bone sarcoma proliferation and bone resorption, leading to investigations about the beneficial effect of anti-resorptive agents on bone sarcoma progression. Thus zoledronate, an N-containing bisphosphonate able to inhibit osteoclasts proliferation and activity, reduces osteosarcoma and Ewing sarcoma primary and metastatic development in corresponding pre-clinical models, disrupting the vicious cycle between tumor growth and osteoclast-mediated bone resorption [22,23,24,25].

Human Mesenchymal Stem Cells (hMSCs) play also a major role in the communication between tumor cells and their microenvironment, as highlighted by recent studies, including increased proliferation and migration of tumor cells. On one hand, bone marrow MSCs promote migration and invasion through direct secretion of several cytokines such as C-X-C motif chemokine ligand 12 (CXCL12) that binds to C-X-C motif chemokine receptor 4 (CXCR4) or CXCR7 on osteosarcoma associated with lower overall patient survival [26,27,28]. Interleukin-6, secreted by MSCs, contributes to promote metastatic dissemination of osteosarcoma [29,30]. On the other hand, MSCs release exosomes that promote tumor growth and metastases [31]. Other types of stem cells such as the stromal adipose-derived mesenchymal stem cells stimulate tumor growth and invasion of osteosarcoma cells, by activating the signal transducer and activator of transcription 3 (STAT3) signaling pathway, resulting in an increase of the expression of matrix metalloproteinase (MMP) 2 and MMP9 [32].

Bone sarcoma is able to modulate the recruitment, expansion and differentiation of immune cells to establish an immunosuppressive microenvironment, allowing tumor development and metastatic dissemination [33]. Regarding innate immunity, bone sarcomas are mainly invaded by TAMs that are initially divided into two categories: anti-tumor M1-polarized macrophages and tumor-promoting M2-polarized macrophages [34]. Macrophages massively invade osteosarcoma, this infiltration being associated with a good prognosis in osteosarcoma patients [35,36]. However, the implication of tumor-promoting M2-polarized macrophages infiltrates in osteosarcoma progression remains controversial. Indeed, M2-type macrophage infiltrates in the tumor have been correlated with a poor prognosis in some studies [36,37]. Moreover, on one hand, an imbalance in favor of M1 macrophages has been observed in patients without metastases [37]. On the other hand, M2-polarized macrophages have been associated with increased tumor growth and metastases in preclinical models of osteosarcoma [38]. However, a recent study based on the most important cohort of osteosarcoma patients from the OS2006 clinical trial has demonstrated that the presence of CD163^+^ M2-polarized macrophages is crucial for the inhibition of osteosarcoma progression [35]. In contrast, macrophages do not seem to play such a role in Ewing sarcoma development and patient survival [39,40]. Moreover, M2-polarized macrophages contribute to the exhaustion of T lymphocytes infiltrating the tumor, reducing their proliferation and secretion of pro-inflammatory cytokines [41]. These results may be associated with the existing metabolic competition between sarcoma cells and infiltrating T lymphocytes. Indeed, excessive consumption of glucose from the microenvironment by tumor cells restricts the consumption of T cells, which show decreased glycolysis and under-production of interferon γ (IFNγ). T cells respond less to stimuli, allowing the tumor to escape from immune control. Thus, metabolic competition between tumor cells and T cells influences tumor progression [42].

Besides cellular components, the physical properties of the TME can influence the behavior of cancer cells. Indeed, mechanical (composition of the extracellular matrix) and chemical stress (hypoxia) increase the motility and capacity of sarcoma cells to metastasize, through increased expression of procollagen-lysine, 2-oxoglutarate 5-dioxygenase 2 (PLOD2), a collagen cross-linker [43]. Hypoxic microenvironment also leads to increased interleukin-6 expression in osteosarcoma cells, which contributes to increased lung metastases [44]. Finally, Ewing sarcoma in response to Wnt3a stimulation can modify the acellular TME by increasing the secretion of proteins involved in the composition of the extracellular matrix [45].

### 2.2. Bone Sarcoma Microenvironment as a Prognostic Marker or Therapeutic Target

Several studies have highlighted the importance of analyzing TME in order to predict patient outcomes. For example, Volchenboum et al. have demonstrated the importance of studying stromal cells in contact with the Ewing sarcoma, to predict patients at high risk of relapse, thanks to a gene set enrichment analysis conducted on human Ewing sarcomas samples by the Children’s Oncology Group (COG) [46]. A strong expression of C-C motif ligand (CCL) 12 produced by infiltrating immune cells is correlated with an increased ratio of CD8^+^/CD4^+^ infiltrating T lymphocytes, leading to a better response to chemotherapy and overall patient survival [47]. In osteosarcoma, tissue micro-arrays analyses showed that the immune infiltrate of primary tumors was enriched with anti-tumor M1-type macrophages for the localized forms (CD68^+^ iNOS^+^, inducible nitric oxide synthase), as compared to metastatic diseases [37]. In addition, metastatic tumors have a higher vascular density than localized tumors, supporting that greater angiogenesis may partly explain metastatic spread.

Osteoclasts and immune cells are the main potential therapeutic targets for osteosarcoma and Ewing sarcoma. Based on preclinical data, the clinical trial OS2006 (NCT00470223, available online: https://clinicaltrials.gov/ct2/show/NCT00470223) has been developed to evaluate the combination of zoledronate with current chemotherapy and surgery in osteosarcoma patients. Unfortunately, the results do not recommend the use of zoledronate as no improvement in event-free survival was observed, zoledronate showing a trend to increase the risk of failure in osteosarcoma patients [48]. Several therapeutic approaches targeting infiltrating immune system are currently in clinical development. For example, some of osteosarcoma and Ewing sarcoma express programmed cell death ligand (PDL)-1 that binds to programmed cell death (PD)-1 on infiltrating T cells, inhibiting their cytotoxic activities and contributing to the local immunosuppression and consequently to the tumor progression [49]. Based on pre-clinical data demonstrating the beneficial effect of PD-1/PDL-1 blockade [50] and correlations between PDL-1 expression and clinical outcomes [51,52], clinical trials evaluating the effects of anti-PD1 antibodies are in progress in osteosarcoma and Ewing sarcoma (Pembrolizumab, NCT02301039 (available online: https://clinicaltrials.gov/ct2/show/NCT02301039) and Nivolumab, NCT02304458 (available online: https://clinicaltrials.gov/ct2/show/NCT02304458)).

## 3. The Canonical Wnt/β-Catenin Signaling Pathway

The Wnt signaling pathway is essential during development and is involved in many cellular processes, such as proliferation, migration, polarization and cellular differentiation. This signaling pathway is therefore finely regulated and mutations or deregulation of the Wnt signaling pathway are often associated with tumor development, chemoresistance, or relapse [53,54,55]. Three Wnt signaling pathways have been described so far: the canonical Wnt pathway or Wnt/β-catenin pathway on which we will focus, and the non-canonical Wnt/PCP (Planar Cell Polarity) and Wnt/Ca^2+^ pathways which are not detailed in this review.

**Figure 1:**
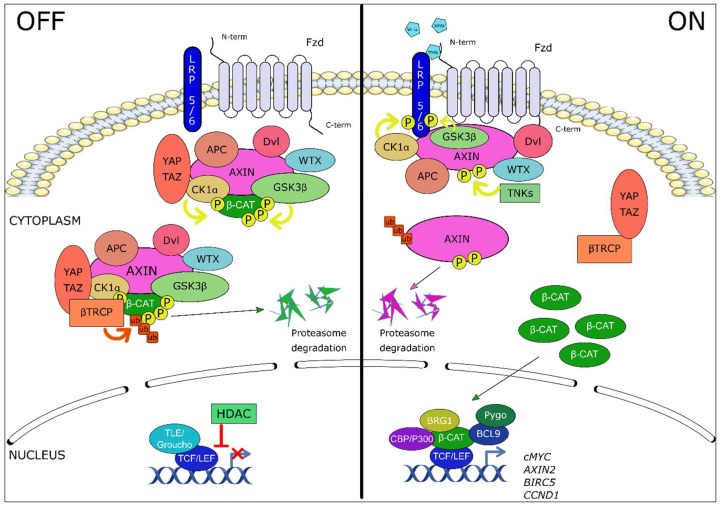
The canonical Wnt/β-catenin signaling pathway. Left panel: in the absence of Wnt ligand, β-catenin is sequestered by a protein complex composed of dishevelled (Dvl), adenomatous polyposis coli (APC), Axin1/2, Wilms tumor gene on X chromosome protein (WTX) and two kinases responsible for the phosphorylation of β-catenin, CK1α (casein kinase 1 alpha) and GSK3β (glycogen synthase kinase 3 beta). Then, YAP/TAZ (yes-associated protein/transcriptional co-Activator with a PDZ-binding domain) proteins recruit β-TrCP (beta-transducin-repeat-containing protein), a ubiquitin ligase responsible for the ubiquitination of β-catenin and its degradation by the proteasome pathway. In the nucleus, the transcriptional proteins of the TCF/LEF family (T-cell factor/lymphoid enhancer-binding factor) interact with the transcriptional repressors groucho/TLE (transducin-like cnhancer of split), recruiting histone deacetylases (HDACs) responsible for repressing transcription. Right panel: Binding of the Wnt ligands to the frizzled (Fzd) receptor and low-density-lipoprotein-related protein 5/6 (LRP5/6) co-receptor complex induces the recruitment of the scaffold protein Dvl to Fzd and leads to LRP5/6 phosphorylation (P) by CK1α and GSK3β kinases. The β-catenin destruction complex is then trapped to the membrane through Axin/Fzd interaction, leading to its inactivation. In parallel, Axin proteins are degraded following poly-ADP-ribosylation by tankyrases (TNKS). Newly synthesized β-catenin accumulates in the cytoplasm and translocates into the nucleus where it interacts with the transcription factors of the TCF/LEF family and with histones modifying co-activators p300 or CREB binding protein (CBP), B cell CLL/lymphoma 9 (BCL-9), brahma-related gene 1 (BRG1), and pygopus. These transcription complexes activate the transcription of target genes such as cMYC, AXIN2, BIRC5 or CCND1.

Wnt ligands are a family of 19 secreted glycoproteins, some of which can bind to one of the 10 receptors of the Frizzled family (Fzd), or to a co-receptor lipoprotein receptor-related protein (LRP)-5 or LRP-6, receptor tyrosine-kinase-like orphan receptor (ROR), related to tyrosine kinase (Ryk), to activate cell signaling. In the absence of Wnt ligands, the Wnt/β-catenin pathway is inactive, β-catenin is sequestered by a protein complex for degradation. This complex is composed of scaffold proteins, dishevelled (Dvl), adenomatous polyposis coli (APC) and Axin1/2 and Wilms tumor gene on X chromosome (WTX) protein [56], and two kinases, casein kinase 1 alpha (CK1α) and glycogen synthase kinase 3 beta (GSK3β) which sequentially phosphorylate β-catenin on Serine 45 (CK1α), and on serine 33 and 37 and threonine 41 (GSK3β). Yes-Associated protein/transcriptional co-activator with a PDZ-binding domain (YAP/TAZ) proteins, part of the complex, then recruit beta-transducin-repeat-containing protein (β-TrCP) an ubiquitin ligase responsible for the ubiquitination of β-catenin [57,58,59,60,61]. β-Catenin is then degraded by the proteasome pathway, and therefore does not play its role as a co-transcription factor. In addition, the transcriptional proteins of the T-cell factor/lymphoid enhancer-binding factor (TCF/LEF) family interact with the transcriptional repressors groucho/transducin-like enhancer of split (TLE), recruiting histone deacetylases (HDACs) responsible for repressing transcription [62] (Figure 1, left panel).

The binding of a Wnt ligand (i.e., Wnt1, Wnt3a) to an Fzd receptor and its co-receptor LRP5/6 activates the canonical Wnt pathway, inducing the recruitment of Dvl on Fzd receptor and its activation. CK1α and GSK3β kinases then phosphorylate the LRP5/6 co-receptor, allowing the recruitment of Axin on LRP5/6 and the inactivation of the β-catenin degradation complex which is trapped to the membrane. Neosynthesized β-catenin is no longer degraded and accumulates in the cytoplasm [63], before being translocated into the nucleus. It interacts with the transcription factors of the TCF/LEF family and with histones modifying co-activators p300 or cAMP-response element binding protein (CREB) binding protein (CBP), B cell CLL/lymphoma 9 (BCL-9), brahma-related gene 1 (BRG1), and pygopus [64,65]. These complexes activate the transcription of target genes such as *AXIN2*, *LEF1*, c*MYC*, *CCND1*, *BIRC5* (Figure 1, right panel).

Numerous molecules are implicated in the regulation of Wnt ligands and the canonical Wnt pathway activation. These include secreted extracellular inhibitors: dickkopf (DKK), secreted-Fzd-related proteins (SFRPs), Wnt inhibitory factor 1 (WIF1), sclerostin (SOST), cerberus, insulin-like growth factor binding protein 4 (IGFBP4). DKK and SOST proteins are competitive receptor and co-receptor antagonists that prevent signaling activation by Wnt ligands. The WIF1, SFRPs (domain Fzd-like C-terminal) and cerberus proteins are able to bind to Wnt ligands preventing their interaction with receptors and co-receptors [65,66].

The canonical Wnt signaling pathway is involved in many cancers, notably in gastrointestinal cancers (colorectal carcinoma, hepatocellular carcinoma, gastric cancer, cholangiocarcinoma, pancreatic ductal adenocarcinoma), hematopoietic cancers (acute myeloid leukemia, chronic myeloid leukemia, acute lymphoblastic leukemia, chronic lymphoblastic leukemia, lymphoma, multiple myeloma), breast cancer, melanoma, and brain tumors such as glioblastoma [67,68]. Its involvement in the tumorigenesis of many cancerous diseases makes it an interesting target for cancer treatment. That is why recent reviews of the literature have described the latest advances in targeting this signaling pathway [55,69,70,71,72,73]. Some Wnt inhibitor molecules are in clinical trials, most of them in Phase I [74,75], although the ubiquitous nature of this signaling pathway and its involvement in various major physiological processes raise the question of the side effects of this strategy [76].

## 4. Key Role of Wnt/β-Catenin Signaling Pathway in Osteosarcoma

The most recent researches on Wnt/β-catenin implication in osteosarcoma deal with metastatic dissemination and recurrence that are associated with poor prognosis for patients. Thus, following a brief description of the implication of the canonical Wnt/β-catenin signaling pathway in osteosarcoma development, this part will focus on these recent studies.

**Figure 2:**
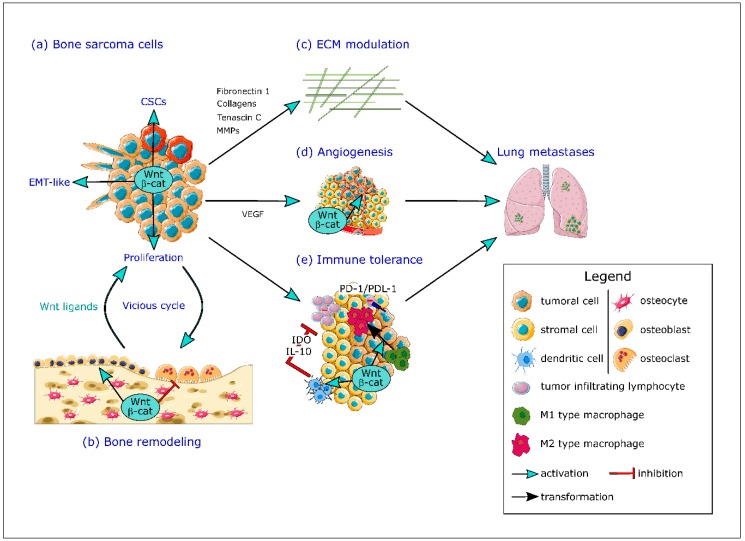
The crucial role of Wnt/β-catenin signaling pathway in multiple steps of bone sarcoma progression and metastatic dissemination. (**a**) The canonical Wnt/β-catenin signaling pathway is able to enhance bone sarcoma cells proliferation, to induce an epithelial-mesenchymal transition (EMT)-like through secretion of fibulin-3 and to promote the acquisition of stem cells properties of bone sarcoma cancer stem cells (CSCs). (**b**) The Wnt/β-catenin pathway participates to the hijacking of the bone microenvironment by the bone sarcoma cells, leading to the establishment of a vicious cycle between bone remodeling and tumor cells proliferation associated with the release of pro-tumoral factors including Wnt ligands from the bone matrix. (**c**) The Wnt/β-catenin pathway promotes the modulation of the extracellular matrix (ECM), increasing secretion of extracellular matrix components such as tenascin C, fibronectin 1 or collagens and stimulates the ECM degradation by upregulation of proteolytic enzymes such as MMPs. (**d**) The Wnt/β-catenin pathway induces an over-expression of vascular endothelial growth factor (VEGF), the most important pro-angiogenic factor and is also able to modulate endothelial cell migration, leading to an increase in tumor-associated angiogenesis. (**e**) The Wnt/β-catenin signaling pathway participates to the establishment of an immune tolerance in the TME, enhancing pro-tumoral M2 macrophages polarization and inhibiting cytotoxic T cell infiltration and functions and inducing resistance to anti-PD1 or anti-PDL-1 therapy. The activation of the Wnt/β-catenin signaling pathway in dendritic cells leads to up-regulation of interleukin-10 (IL-10) and indoleamine 2,3-dioxygenase 1 (IDO) secretion leading to an inhibition of tumor-infiltrating lymphocytes (TILs) cytotoxic properties. By targeting both osteosarcoma and Ewing sarcoma cells and the bone TME, the Wnt/β-catenin signaling pathway participates to the disease progression and the establishment of lung metastases.

Canonical Wnt/β-catenin signaling pathway has been involved in the development of osteosarcoma but current knowledge remains unclear because of the high complexity of this pathway. Many studies support an aberrant activation of the canonical Wnt signaling pathway in osteosarcoma cells which seems to play a crucial role in tumorigenicity as well as metastatic dissemination [77,78]. As an example, two recent analyses on patient samples described a high β-catenin level in osteosarcoma tissues compared to adjacent healthy tissues associated with poor prognosis and lung metastatic dissemination [79,80] (Figure 2a). However, other studies highlighted that inactivation of the Wnt/β-catenin pathway plays a key role in osteosarcoma development [81,82]. In particular, frequent deletions of genes involved in the Wnt signaling pathway genes have been described in osteosarcoma patients [83]. In this context, Shimozaki et al. showed that pharmacological inhibition or depletion of GSK3β by siRNA decreases osteosarcoma cell proliferation despite an increased nuclear translocation of β-catenin and expression of its target genes supporting the tumor suppressor role of β-catenin [84]. However, to our knowledge, no additional recent study seems to support this second hypothesis.

A key issue of osteosarcoma metastasis is the epithelial-mesenchymal transition (EMT)-like multi-step process, based on a transformation from an epithelial to a mesenchymal phenotype, promoting the invasive capacities of cancer cells. EMT is characterized by repression of key components of the intercellular junctions such as E-cadherin associated with an increase in mesenchymal markers including N-cadherin, vimentin and fibronectin. This modulation of gene expression is regulated by different transcription factors such as Snail-1, Snail-2, ZEB-1, ZEB-2 or Twist [85]. Despite the fact that osteosarcoma derives from cells of mesenchymal origin, an overexpression of EMT transcription factors such as Snails, ZEBs or Twist has been associated to increased invasive properties of osteosarcoma cells promoting the formation of metastases [86,87,88,89]. Transforming growth factor beta (TGFβ) seems to be the main inducer of EMT in tumor cells [90], but cooperates with other signaling pathways including the Wnt/β-catenin pathway to induce complete EMT [91]. Recent studies focusing on molecules implicated in EMT in osteosarcoma highlighted the implication of β-catenin in this biological process. Thus, Tian et al. have recently demonstrated that bone morphogenetic protein (BMP)-2 promotes EMT of osteosarcoma cells associated with enhanced motility and invasiveness through the Wnt/β-catenin signaling pathway [92]. Similarly, Fibulin-3, an extracellular glycoprotein, enhances EMT of osteosarcoma cells, associated with Wnt/β-catenin pathway activation [93] (Figure 2a). Some other molecules implicated in osteosarcoma development and metastasis, such as ubiquitin-specific protease 7 and N-terminal truncated form of carboxypeptidase seem to exert their effects through induction of EMT in osteosarcoma cells by activating the Wnt/β-catenin signaling pathway [94,95]. These numerous recent studies demonstrate the interest to target Wnt/β-catenin pathway in order to inhibit EMT in osteosarcoma cells.

EMT can also promote tumor cells to develop stem cell characteristics and consequently actively participate in the cancer stem cell (CSC) phenotype acquisition [96]. CSCs (also known as tumor-initiating cells or TIC) constitute a subpopulation of tumor cells with properties to self-renew and are responsible for tumor initiation and progression and, thanks to their chemotherapy resistance, seem to drive recurrences and metastases. CSCs are characterized by persistent activation of highly conserved signal transduction pathways including notch, hedgehog and Wnt pathways [97] and some evidence support the presence of CSCs in osteosarcoma and their contribution to metastatic chemotherapy-resistant properties [98,99]. Martin-Neves et al. have described a constitutive activation of the Wnt/β-catenin signaling in osteosarcoma CSCs rather than in parental osteosarcoma cells [100]. More recently, Liu et al., highlighted that β-catenin positively regulates osteosarcoma stem cells properties in vitro [79] (Figure 2a). These results support evidence that a therapy against the Wnt/β-catenin pathway in osteosarcoma could allow to target CSCs and potentially improve treatment for metastatic and chemotherapy-resistant patients.

New insights about the role of long non-coding RNA (lncRNA) and micro RNA (miR) in osteosarcoma progression have also recently emerged. On one hand, the overexpression of a number of miR has been demonstrated in osteosarcoma cells leading to increased osteosarcoma growth and metastasis. Most of these miRs downregulate antagonists of the Wnt/β-catenin signaling pathway. As an example, miR-552-5p is overexpressed in osteosarcoma tissues and cell lines compared to healthy tissue and osteoblasts respectively. Knockdown of this miR decreases osteosarcoma proliferation and metastasis by directly inhibiting WIF1 [101]. On the other hand, some miRs that are able to restrain the expression of key intermediates or regulators of the Wnt/β-catenin signaling pathway have been described to be downregulated, causing an aberrant activation of this pathway in osteosarcoma cells. Very recent studies described a decrease in miR-377, miR-873, miR-758 and miR-885-5p in osteosarcoma tissues and/or cell lines, leading to increased primary tumor and metastatic development in association with activated Wnt/β-catenin signaling pathway [102,103,104,105]. Other studies deal with lncRNA such as lncRNA AWPPH which levels are increased in osteosarcoma tissues compared to healthy counterparts and promote tumor proliferation and metastasis through activation of the Wnt/β-catenin pathway [106].

In addition to β-catenin, other modulators of the canonical Wnt signaling pathway, in particular, Wnt antagonists, are affected during osteosarcoma primary and metastatic development, but have already been described in several reviews and will not be developed here [107,108].

## 5. Involvement of Wnt/β-Catenin Signaling Pathway in Ewing Sarcoma

The involvement of the Wnt signaling pathway in Ewing sarcoma is less described than in osteosarcoma and seems to be controversial. First, no recurrent mutations in the Wnt/β-catenin signaling pathway have been reported in Ewing sarcoma but the activation of this pathway has been demonstrated in aggressive sarcoma, the bone microenvironment being the source of Wnt ligands. Moreover, this activation can be potentiated through the R-spondin (RSPO)/leucine rich repeat containing G protein-coupled receptor 5 (LGR5)/β-catenin axis. Indeed, LGR5 is highly expressed in aggressive Ewing sarcoma and CSCs and the binding of RSPO-2 on LGR5 was able to potentiate Wnt3a-induced canonical Wnt signaling. However, it seems that an important variability in Wnt responsiveness exists among Ewing sarcoma cells based on the differential expression of LGR5 and the availability of Wnt and RSPO ligands in the tumor microenvironment [109]. The proliferation of Ewing sarcoma cells is not affected by the Wnt/β-catenin pathway activation [110]. However, this signaling pathway appears to play a crucial role in the cellular motility of Ewing sarcoma cells [111]. Thus, the activation of the Wnt/β-catenin signaling pathway in Ewing sarcoma cells induces, on one hand, the formation of neurite outgrowth and, on the other hand, changes in transcriptional profile, leading to the acquisition of a more aggressive metastatic phenotype by tumor cells [109,112]. To go further, molecular mechanisms have been explored. Surprisingly, the activation of the Wnt/β-catenin pathway partially antagonizes the transcriptional function of the fusion protein EWS/FLI1 (found in the majority of Ewing sarcomas), resulting in an up regulation of metastasis-promoting genes that are normally repressed by EWS/FLI1 and consequently, the acquisition of a more migratory phenotype. Inversely, some studies have shown that EWS/FLI1 inhibits β-catenin dependent transcription through a direct interaction between EWS/FLI1 and the transcription factor LEF1, inhibiting the interaction between β-catenin and LEF1 in Ewing sarcoma-cell lines [113]. To sum up, an inverse relationship between the expression of EWS/FLI1 and TCF/LEF target genes is observed in Ewing sarcoma cells [109,113].

The analysis of the secretome of Wnt3a-activated Ewing sarcoma cells has revealed an increased secretion of proteins implicated in the composition and the structure of the extracellular matrix. In details, Wnt3a-activated cells present an upregulation of the signaling pathways involved in the communication between the tumor and its microenvironment, such as integrin linked kinase, focal adhesion kinase, cadherin angiogenesis, TGFβ and matrix metalloproteinase pathways [45].

Finally, the effects of WNT974, a Porcupine protein inhibitor involved in the palmitoylation of Wnt ligands, an indispensable process for Wnt secretion has been investigated in Ewing sarcoma. The WNT974-mediated inhibition of endogenous Wnt ligands secreted by tumor cells decreases the migration of Ewing sarcoma cells in vitro, downregulating the expression of genes involved in cell migration. In addition, WNT974 appears to reduce the development of metastatic disease in vivo in a murine subcutaneous xenograft model and to delay the time of first lung metastasis establishment. WNT974 inhibits Wnt signaling pathways (canonical and non-canonical) and cell migration and dissemination, therefore demonstrated the role of the Wnt signaling pathway during Ewing sarcoma metastatic spread [114].

## 6. Wnt/β-Catenin Pathway and the Bone Tumor Microenvironment

As described in the first part of the review, the bone TME is a complex and dynamic environment composed of both cellular and non-cellular components. This TME which includes bone, vascular and immune “niches” plays a key role in initiation, primary development and metastasis of osteosarcoma and Ewing sarcoma [115]. This part will focus on the implication of the canonical Wnt/β-catenin signaling pathway in these three “niches” of the bone TME.

### 6.1. Wnt/β-Catenin Pathway and Bone Remodeling

Bone remodeling results from a balance between osteoclasts-mediated bone resorption and osteoblasts-promoted bone formation. Osteocytes, trapped in the bone matrix, also contribute to the regulation of osteoblast and osteoclast functions during bone remodeling. In this context, the Wnt/β-catenin signaling pathway is able to modulate both osteoblast and osteoclast differentiation and activity. Indeed, osteoblasts and their precursors, osteocytes and osteoclasts are able to secrete various Wnt ligands which participate in the control of bone remodeling [116,117,118]. Briefly, the Wnt/β-catenin signaling pathway directly enhances osteoblast differentiation and bone formation whereas indirectly represses osteoclast differentiation and bone resorption through the production of osteoprotegerin by osteoblasts and osteocytes. Nevertheless, osteoclasts are also affected by Wnt ligands given that β-catenin activation enhances the proliferation of osteoclast precursors but inhibits osteoclastogenesis at a later stage [66,116,119].

The early-stage development of osteosarcoma and Ewing sarcoma initiates a dysregulation of the bone remodeling in favor of an exacerbated bone resorption, leading to the release of pro-tumorigenic factors initially trapped into the bone tissue (Figure 2b). Nevertheless, the implication of the Wnt/β-catenin signaling pathway in tumor cell-induced bone osteolysis has mainly been described in bone metastasis rather than in bone sarcoma. Interestingly, in breast cancer bone metastases, the Wnt/β-catenin signaling pathway seems to be active inside the tumor with overexpression of canonical Wnt ligands (Wnt2 and Wnt8b), maintaining tumor cell proliferation. Conversely, Wnt2 and Wnt8b are downregulated at the tumor/bone interface while Wnt antagonists WIF1 and SFRP4 are overexpressed and could be responsible, at least in part, of suppression of osteoblast proliferation and enhanced bone destruction by osteoclasts [120]. Moreover, the DKK1 overexpression has been described in osteolytic breast cancer and could promote osteoclastogenesis and bone resorption at the site of metastasis [121]. To our knowledge, no studies compare the activation of the canonical Wnt signaling pathway between the bone sarcoma core and the bone/tumor interface.

An important step in the metastatic process of bone sarcoma is the degradation of the extracellular matrix related to upregulation of proteolytic enzymes such as MMPs. Several studies have highlighted that the invasive capacities of osteosarcoma cells are correlated with MMPs expression, particularly MMP-2 and MMP-9 [122]. Moreover, MMP-2 and/or MMP-9 expression can be associated with the development of metastasis, poor prognosis and resistance to chemotherapy in osteosarcoma patients [123,124,125]. It is also well established that MMP2 and MMP9 are expressed in Ewing sarcoma and closely associated with Ewing sarcoma invasion and metastasis [126,127]. β-catenin gene silencing by siRNA reduced invasion and motility of the U2OS human osteosarcoma cell line associated with inhibition of membrane type 1 metalloproteinase (MT1-MMP) expression. On one hand, MT1-MMP directly degrades extracellular matrix components such as type II collagen or fibronectin and, on the other hand, activates by cleavage other members of the MMP family such as MMP-2. Moreover, a dominant-negative soluble LPR5 reduces the expression of MMP-2 and MMP-14, consistent with a decrease in invasive properties of the human SaOS2 osteosarcoma cell line [128]. Thus, these results suggest that β-catenin inhibition may decrease osteosarcoma invasion through downregulation of MMPs expression and activation [129,130,131]. In Ewing sarcoma, the implication of the Wnt/β-catenin signaling pathway does not seem to be predominant for the expression and activation of MMPs. However, in Ewing sarcoma, Wnt/β-catenin activation-induced expression of tenascin C, a secreted extracellular matrix protein that plays a key role during metastasis establishment in different types of cancer [132]. High level of tenascin C contributes to the Ewing sarcoma metastatic phenotype, modulating tumor microenvironment [109]. Activation of the canonical Wnt pathway also leads to increased secretion of the extracellular matrix components such as fibronectin or collagens, that alter the biochemical and structural properties of the bone TME and can modulate the metastatic process of Ewing sarcoma [45] (Figure 2c).

### 6.2. Wnt/β-Catenin Pathway, Angiogenesis and Hypoxia

Angiogenesis is a complex biological process that leads to new blood vessel development and plays a key role in primary tumor progression and metastatic potential of several cancers including bone sarcoma. Stages of angiogenesis include the proliferation and migration of endothelial cells to form a vessel sprout and then the recruitment of pericytes or smooth muscle cells to produce a mature and functional blood vessel [133]. This process is necessary for tumor growth allowing the supply of nutrients and metastatic dissemination, providing an intravasation site for cancer cells [133,134]. Vascular endothelial growth factor (VEGF) is the most important pro-angiogenic factor involved in the formation of new functional vessels in tumors through recruitment and differentiation of endothelial progenitor cells [135].

Osteosarcoma patients with high VEGF expression levels exhibit both lower disease-free survival and lower overall survival [136]. Similarly, a genetic amplification of the *VEGFA* gene was detected in osteosarcoma and significantly associated with high microvessels density and low patient disease-free survival [137]. Several studies have also highlighted the importance of VEGF in angiogenesis of Ewing sarcoma. Indeed, high levels of VEGF have been detected in the serum of Ewing sarcoma patients and correlated with increased microvascular density and poor patient outcomes [135,138,139]. In particular, Ewing sarcoma cells produce several VEGF isoforms including soluble spliced isoform of VEGFA, VEGF-165, which promotes the recruitment of bone marrow derived cells. These cells can differentiate into both tumor-associated pericytes and endothelial cells, contributing to the new vasculature of Ewing sarcoma [140,141].

Regarding the role of the Wnt/β-catenin signaling pathway in angiogenesis, transcriptional regulation of VEGF by the β-catenin/TCF complex has been demonstrated, involving TCF binding sites in the *VEGF* gene promoter. Moreover, defects in APC, resulting in constitutively active β-catenin, induce the over-expression of VEGF [142,143]. Furthermore, the SFRPs proteins are a family of soluble Wnt inhibitors that act either through direct binding to Wnt proteins and preventing its interaction with Fzd receptors or through direct interaction with Fzd receptors [144]. First, endothelial cells seem to have a sequential response to SFRP-1 including an initial inhibition of angiogenesis by antagonizing the canonical Wnt signaling pathway followed by an activation of the non-canonical Wnt/PCP signaling pathway that promotes angiogenesis [145,146]. Second, a study has demonstrated that SFRP-4 induces a decrease in endothelial cell migration correlated with a reduction in tumor-associated angiogenesis in a mouse model of ovarian cancer [147]. All these studies highlighted the role of the canonical Wnt/β-catenin signaling pathway in promoting angiogenesis (Figure 2d).

Only a few studies deal with the implication of the canonical Wnt/β-catenin signaling pathway in bone sarcoma-associated angiogenesis. Nevertheless, a study has described that naked cuticle homolog 2 (NKD2), a negative regulator of Wnt signaling, downregulates the expression of genes involved in the vasculature development of osteosarcoma [148], suggesting a pro-angiogenic role of the Wnt/β-catenin pathway in osteosarcoma. To our knowledge, the implication of this pathway on Ewing sarcoma-associated angiogenesis has not been described yet.

It is well recognized that hypoxia is an important regulator of angiogenesis during tumor development [149]. A major regulator of hypoxia is the transcription factor hypoxia inducible factor 1 (HIF-1), composed of the oxygen regulated HIF-1α subunit and the constitutively expressed HIF-1β. Under the hypoxic TME, HIF-1 directly activates the transcription of pro-angiogenic genes including VEGF to promote angiogenesis [150,151] as demonstrated in osteosarcoma and Ewing sarcoma [152]. Thus, HIF-1α silencing by siRNA reduces VEGF production by osteosarcoma cells in vitro and inhibits angiogenesis in a mouse model of osteosarcoma [153].

Besides its regulation of angiogenesis, hypoxia has been directly correlated to bone sarcoma progression, metastasis and patient prognosis. Thus, hypoxia enhances the expression and the transcriptional function of the fusion protein EWS/FLI1 in a HIF-1α dependent-manner in Ewing sarcoma cells and consequently increases their invasiveness [154], HIF-1α is also able to promote osteosarcoma cell proliferation and migration through activation of several signaling pathway including Akt and STAT3 signaling pathways [155,156]. Moreover, the expression of HIF-1α has been correlated to the development of lung metastasis associated with poorer survival in osteosarcoma patients [157,158,159].

The implication of the Wnt/β-catenin signaling pathway in hypoxia has mainly been reported in carcinoma. As an example, hypoxia promotes β-catenin nuclear localization related to a more invasive phenotype in colorectal adenocarcinoma cells [160]. Moreover, β-catenin directly interacts with HIF-1α to promote HIF-1 mediated transcription allowing an enhanced survival and adaptation to hypoxia of colon cancer cells [161]. Although the canonical Wnt/β-catenin signaling pathway seems to play a crucial role in hypoxia, only a few studies deal with its implication in the hypoxic TME of bone sarcoma. The most relevant one has described that inhibitors of the Wnt signaling pathway re-sensitizes osteosarcoma cells to doxorubicin under hypoxic conditions [162].

### 6.3. Wnt/β-Catenin Pathway and the Immune System

The immune microenvironment of bone sarcomas is highly heterogeneous composed of effectors of the innate immunity such as natural killer (NK) cells, dendritic cells (DC) and tumor-associated macrophages (TAMs) and cytotoxic T cells which drive adaptive immunity [33,35]. Nevertheless, immune and inflammatory infiltrates remain lower in Ewing sarcoma, limiting the development of immunotherapy [163].

Regarding innate immunity, bone sarcomas are mainly invaded by TAMs which have been associated with a good prognosis in osteosarcoma patients. However, as described above, it remains difficult to conclude about the role of M2-polarized macrophages in osteosarcoma progression, probably because of the difficulty to clearly identify the different TAMs subtypes in tumors and by the fact that, in contrast to the binary M1/M2 subtype, TAMs correspond to several distinct populations that often share features of both subtypes [164]. In Ewing sarcoma, a first study has initially described a correlation between macrophages infiltrate and poor prognosis but a second one did not observe any correlation between CD68^+^ macrophages and survival [39,40]. The canonical Wnt/β-catenin signaling pathway seems to drive the polarization of macrophages in cancers. Indeed, hepatic tumor cells produce Wnt ligands that enhance M2 macrophages polarization through the Wnt/β-catenin signaling pathway, resulting in increased primary and metastatic tumor development [165] (Figure 2e).

T lymphocytes in cancer, also known as tumor-infiltrating lymphocytes (TIL), correspond to the second most important immune cells in osteosarcoma [33,166]. TILs had higher cytotoxic capacities against tumor cells than peripheral lymphocytes but immunomodulatory molecules present in the TME prevent their expansion and activation. In particular, ligands of the B7 family are usually expressed by tumor cells in order to modulate T lymphocytes activities [167]. Osteosarcoma cells express HERV-H LTR-associating 2 (HHLA2), PDL-1 and B7-H3, three members of the B7 proteins that contribute to local immunosuppression. HHLA2 expression has been associated with metastatic properties of osteosarcoma cells and poor survival of osteosarcoma patients [168,169]. The binding of PDL-1, expressed by osteosarcoma cells to PD-1 on TILs induces the inhibition of their cytotoxic activities, contributing to local immunosuppression and consequently to tumor development. On the contrary, Ewing sarcoma does not constitutively express PDL-1 although its expression could be induced under inflammatory conditions [170]. Thus, only one case of the response of Ewing sarcoma to the blockade of PD-1/PDL-1 interaction has been described [171]. Finally, osteosarcoma cells also express B7-H3 which is correlated with tumor aggressiveness and metastasis [172,173]. In this context, Wnt/β-catenin pathway activation in the TME seems to inhibit T cell infiltration and functions in several cancers [174,175]. Moreover, the active Wnt/β-catenin signaling pathway has been associated with T cell exclusion leading to resistance to anti-PDL-1 therapy in human melanoma [176] (Figure 2e). Similarly, β-catenin activation enhances immune evasion and induces resistance to anti-PD1 therapy in hepatocellular carcinoma [177].

Another important point is that cross regulations between TAMs and TILs play a key role in osteosarcoma development. Thus, M2-polarized macrophages promote the suppression of TILs in osteosarcoma and, conversely, anti-PD1 therapy targeting TILs is able to stimulate a transition from M2 macrophages to M1 macrophages, leading to a decrease in lung metastases [41,178].

Finally, it seems important to note that, besides TAMs and TILs, the Wnt/β-catenin pathway is able to modulate the proliferation, recruitment, and/or functions of the different effectors of the immune system and to regulate multiple aspects of the immune cells/tumor crosstalk. As an example, Wnt ligands secreted by tumor cells can activate the Wnt/β-catenin signaling pathway in DC, leading to up regulated interleukin-10 and indoleamine 2,3-dioxygenase 1 secretion. This results in an increase in regulatory T cells inside the tumor and consequently an inhibition of TILs cytotoxic properties [179].

## 7. Conclusions

The canonical Wnt/β-catenin signaling pathway plays a crucial role during the different steps of bone sarcoma growth and the metastatic process, although its involvement has been more studied in osteosarcoma than in Ewing sarcoma. This pathway seems to promote bone sarcoma development through both a direct effect on bone sarcoma cells and an indirect modulation of the bone TME cell activities. Thus, aberrant activation of the canonical Wnt signaling pathway in osteosarcoma cells seems to be highly involved in cell proliferation, induction of an EMT-like process, acquisition of stem cells properties of osteosarcoma CSCs as well as metastatic dissemination. The Wnt/β-catenin pathway activation does not affect the proliferation of Ewing sarcoma cells but appears to play a role in the cellular motility of these cells. The Wnt/β-catenin pathway also participates to the hijacking of the bone TME by the bone sarcoma cells, promoting modulation of the ECM to allow the tumor cells invasiveness and inducing immune tolerance and angiogenesis in the TME. This results in an increase in metastatic dissemination.

The crucial role of the canonical Wnt/β-catenin signaling pathway is not restricted to bone sarcoma. That is why different therapeutic strategies targeting the Wnt/β-catenin signaling pathway in cancers have been developed. Corresponding clinical trials have been recently described in details in two reviews [67,174]. Briefly, they include inhibitors of porcupin, the enzyme responsible for palmitoylation of Wnt ligands, a necessary process to allow their extracellular secretion (LGK974 or WNT974), anti-Fzd antibodies (OMP18R5) or inhibitors of the β-catenin transcriptional complex (PRI724), mainly evaluated in patients with solid tumors.

The complexity of the Wnt signaling offers multiple targets to reduce β-catenin activation in tumor cells. However, better knowledge about the role of Wnt/β-catenin in each tumor cell type, in particular, osteosarcoma and Ewing sarcoma, is necessary to safely target this pathway. It is also necessary to focus on crosstalks between the Wnt/β-catenin and other signaling pathways during bone sarcoma growth and metastatic processes.

## Abbreviations

APC	Adenomatous Polyposis Coli
BCL-9	B cell CLL/lymphoma 9
β-TrCP	beta-Transducin-repeat-Containing Protein
BRG1	Brahma-related gene 1
CBP	CREB (cAMP-Response Element Binding protein) binding protein
CCL	C-C motif ligand
CK1α	Casein Kinase 1 alpha
CSC	Cancer Stem Cell
CXCL12	C-X-C Motif Chemokine Ligand 12
CXCR	C-X-C Motif Chemokine Receptor
DC	Dendritic cell
DKK	Dickkopf
Dvl	Dishevelled
EMT	Epithelial-Mesenchymal Transition
ETS	E26 Transformation-Specific
Fzd	Frizzled
GSK3β	Glycogen synthase kinase 3 beta
HDAC	histone deacetylase
HHLA2	HERV-H LTR-associating 2
HIF-1	Hypoxia Inducible Factor 1
IFNγ	Interferon γ
IGFBP4	Insulin-like Growth Factor Binding Protein 4
iNOS	inducible Nitric Oxide Synthase
LEF	Lymphoid Enhancer-binding Factor
LGR5	Leucine Rich Repeat Containing G Protein-Coupled Receptor 5
lncRNA	long non-coding RNA
LRP	Lipoprotein Receptor-related Protein
miR	miRNA
MMP	Matrix Metalloproteinase
MSC	Mesenchymal Stem Cell
MT1-MMP	Membrane type 1 metalloprotease
NK	Natural killer
NKD2	Naked cuticle homolog 2
PCP	Planar Cell Polarity
PD-1	Programmed cell Death 1
PDL-1	Programmed cell Death Ligand 1
PLOD2	Procollagen-Lysine, 2-Oxoglutarate 5-Dioxygenase 2
ROR	Receptor tyrosine-kinase-like Orphan Receptor
RSPO	R-spondin
Ryk	Related to tyrosine kinase
SFRP	Secreted-Fzd-Related Protein
SOST	Sclerostin
STAT3	Signal transducer and activator of transcription 3
TAF	Tumor Associated Fibroblast
TAM	Tumor Associated Macrophage
TAZ	Transcriptional co-Activator with a PDZ-binding domain
TCF	T-Cell Factor
TGFβ	Transforming Growth Factor beta
TLE	Transducin-Like Enhancer of split
TIL	Tumor Infiltrating Lymphocyte
TME	Tumor MicroEnvironment
VEGF	Vascular Endothelial Growth Factor
WIF1	Wnt Inhibitory Factor 1
WTX	Wilms tumor gene on X chromosome
YAP	Yes-Associated Protein
